# Increased pain in veterans with cancer—time to re-evaluate opioid prescribing practices?

**DOI:** 10.1093/jncics/pkae024

**Published:** 2024-04-11

**Authors:** Vikram Jairam

**Affiliations:** Department of Radiation Oncology, Sutter Medical Group, Sacramento, CA, USA

The United States is in the middle of an opioid epidemic. The number of opioid-related overdose deaths in 2021 was at its highest in any year, claiming more than 80 000 lives (1). This is primarily due to an increase in synthetic opioids, such as fentanyl, responsible for 70 000 overdose-related deaths. Meanwhile, prescription opioid deaths rose steadily from 2000 and peaked in 2017, now having decreased and stabilized since then. This is in large part due to increased regulation and scrutiny of opioid prescribing, with initiatives such as prescription drug monitoring programs, temporal limits on initial opioid prescriptions, insurance-related prior authorizations, and national clinical practice guidelines (2,3). In addition, there has been a marked change in attitudes of prescribers toward opioid prescribing, with a heightened concern for high-risk use and misuse (4). As a result, there was a 20% decrease in opioid prescribing nationwide between 2013 and 2017 (5).

Veterans represent a unique population who are particularly prone to experiencing chronic pain compared with the general population, in part due to the physical and mental demands of military service (6). In turn, high-risk opioid use is especially prevalent in this population (7). In 2013, the Veterans Health Administration (VHA) launched the Opioid Safety Initiative, aimed to ensure veterans are prescribed opioids in a safe and effective manner. However, there is a paucity of long-term data describing patterns of opioid prescribing and how this has impacted pain control. This question is of particular interest in patients with cancer and cancer survivors, who have unique pain management needs that may be impacted by well-intentioned initiatives curbing opioid prescribing.

To study this, Mudumbai and colleagues explored patterns of opioid prescribing and pain in a population of veterans at a tertiary VHA system between 2015 and 2021 (8). Patient demographic and medical history was extracted from electronic health records along with opioid prescription data. Opioid prescriptions of 30, 60, and 90 consecutive days along with average morphine milligram equivalents (MME) per year were calculated. Cancer incidence was obtained from a linked database and included all cancer diagnoses except benign lesions and nonmelanoma skin cancers. The highest pain score for a patient recorded in a given year was used to determine pain level. Patterns among patients with and without cancer were compared.

As expected, the authors found patients with cancer were more likely to receive an opioid prescription and had a higher average MME compared to noncancer patients. Perhaps the most salient findings of the study pertain to the temporal trends in opioid prescribing and incidence of severe pain. The study reports significant decreases in opioid prescribing with an average 60% decrease per year in MME in both cohorts. This decrease accelerated more in the last 2 years of the study in patients with cancer compared to noncancer patients. When pain scores were evaluated, there was a concerning increase in the incidence of severe pain from 29.5% to 31.9% among patients with cancer, whereas this rate remained steady (27.4%) among noncancer patients.

This study raises a critical issue regarding the appropriateness of shifting prescribing patterns among patients with cancer. Although it is known that opioid prescribing and utilization have decreased among cancer survivors (5,10,11), there is a growing body of data to suggest this paradigm shift may not reflect appropriate changes in clinical practice. Multiple studies have found that reduced opioid prescribing in patients with cancer has led to worse pain control (11), is not tailored to patients at highest risk of misuse (12), and is more prevalent among racial and ethnic minorities (13,14). Notably, this study by Mudumbai and colleagues is unique in evaluating pain in the outpatient setting and including a noncancer comparison group. It suggests that efforts toward reduced opioid prescribing are leading to higher levels of uncontrolled pain among patients with cancer. Indeed, there is growing concern that tighter regulations and heightened scrutiny associated with opioid prescribing has inadvertently impacted pain control among cancer survivors (15-17). In 2022, this led the Centers for Disease Control and Prevention to amend its 2016 clinical practice guidelines for opioid prescribing in patients with pain to more explicitly emphasize individualized decision-making and exclude patients with cancer-related pain (18).

As the authors note, there are some limitations of this study, as well as questions this study raises for further exploration. First, this was conducted at a single VHA tertiary care hospital and may not reflect practice patterns among all veterans. Efforts are underway to expand this study to a broader range of VHA centers. Second, although the study suggests a potential correlation between decreasing opioid prescribing and increasing pain scores, there may be other etiologies contributing to the increase in severe pain. For example, improvements in life expectancy and cancer treatments have resulted in more cancer survivors, particularly elderly patients, living longer and with lasting side effects from their disease or treatments, including chronic pain (19). Adults aged 85 years and older are the fastest-growing group of cancer survivors, and it is known that cancer-related pain is woefully undertreated in elderly patients (20). Future studies could explore more granular measures of pain and particular subgroups of patients with cancer. For example, an analysis of the average or median pain scores for a patient within a given year may provide a meaningful assessment of overall symptom control and minimize the impact of outlier events. In addition, a study of pain scores in patients whose opioid prescriptions remained steady over time may help clarify the association between opioid prescribing and pain control. Finally, it would be informative to understand how opioid prescribing patterns and pain scores differ among patients with localized vs metastatic disease, as these comprise separate populations with different pain management needs.

## Data Availability

No new data were generated or analyzed for this editorial.
